# Supplementary material for: The interactome of intact mitochondria by cross-linking mass spectrometry provides evidence for coexisting respiratory supercomplexes
Source: Mol Cell Proteomics. 2017 Dec 8;17(2):216–32. doi: 10.1074/mcp.RA117.000470 (PMC5795388; doi:10.1074/mcp.RA117.000470)
Supplement: Supplemental Data [file supp_RA117.000470_133922_0_supp_23978_fzffwf.zip › spectra_annotation/mito_DR_spectra_annotation/229-1-7-1-2-1.pdf]

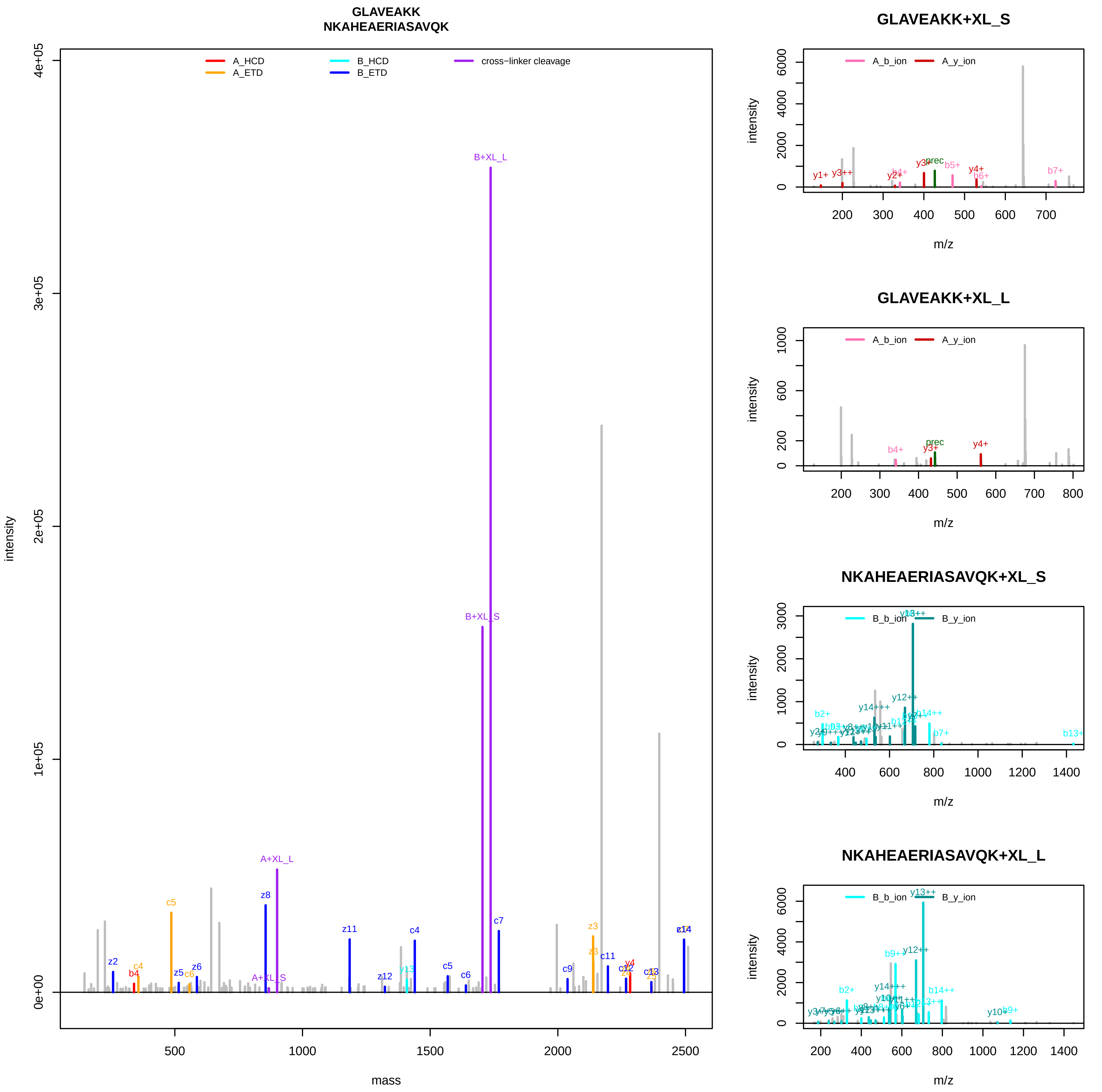
GLAVEAKK
NKAHEAERIASAVQK
A_HCD
A_ETD
B_HCD
B_ETD
cross−linker cleavage
B+XL_L
B+XL_S
A+XL_L
A+XL_S
intensity
mass
0e+00
1e+05
2e+05
3e+05
4e+05
500
1000
1500
2000
2500
z2
b4
c4
c5
z5
c6
z6
z8
z11
z12
y13
c4
c5
c6
c7
c9
z3
c11
y4
c12
c13
z14
GLAVEAKK+XL_S
A_b_ion
A_y_ion
y1+
y3++
b4+
y2+
y3+
prec
b5+
y4+
b6+
b7+
m/z
GLAVEAKK+XL_L
A_b_ion
A_y_ion
b4+
y3+
prec
y4+
m/z
NKAHEAERIASAVQK+XL_S
B_b_ion
B_y_ion
y13++
y12++
y14+++
b2+
b14++
b7+
b13+
m/z
NKAHEAERIASAVQK+XL_L
B_b_ion
B_y_ion
y13++
b9++
y12++
b2+
y14+++
b14++
y10++
b9+
m/z
